# The taxonomic status of the endangered thin-spined porcupine, *Chaetomys subspinosus *(Olfers, 1818), based on molecular and karyologic data

**DOI:** 10.1186/1471-2148-9-29

**Published:** 2009-02-03

**Authors:** Roberto V Vilela, Taís Machado, Karen Ventura, Valéria Fagundes, Maria José de J Silva, Yatiyo Yonenaga-Yassuda

**Affiliations:** 1 Departamento de Genética e Biologia Evolutiva, Instituto de Biociências, Universidade de São Paulo, Rua do Matão, 277, Cidade Universitária, São Paulo, SP, Brazil; 2 Laboratório Especial de Ecologia e Evolução, Instituto Butantan Avenida Dr Vital Brazil, 1500, São Paulo, SP, Brazil; 3 Departamento de Ciências Biológicas, Centro de Ciências Humanas e Naturais, Universidade Federal do Espírito Santo, Avenida Marechal Campos, 1468, Maruípe, Vitória, ES, Brazil

## Abstract

**Background:**

The thin-spined porcupine, also known as the bristle-spined rat, *Chaetomys subspinosus *(Olfers, 1818), the only member of its genus, figures among Brazilian endangered species. In addition to being threatened, it is poorly known, and even its taxonomic status at the family level has long been controversial. The genus *Chaetomys *was originally regarded as a porcupine in the family Erethizontidae, but some authors classified it as a spiny-rat in the family Echimyidae. Although the dispute seems to be settled in favor of the erethizontid advocates, further discussion of its affinities should be based on a phylogenetic framework. In the present study, we used nucleotide-sequence data from the complete mitochondrial cytochrome *b *gene and karyotypic information to address this issue. Our molecular analyses included one individual of *Chaetomys subspinosus *from the state of Bahia in northeastern Brazil, and other hystricognaths.

**Results:**

All topologies recovered in our molecular phylogenetic analyses strongly supported *Chaetomys subspinosus *as a sister clade of the erethizontids. Cytogenetically, *Chaetomys subspinosus *showed 2n = 52 and FN = 76. Although the sexual pair could not be identified, we assumed that the X chromosome is biarmed. The karyotype included 13 large to medium metacentric and submetacentric chromosome pairs, one small subtelocentric pair, and 12 small acrocentric pairs. The subtelocentric pair 14 had a terminal secondary constriction in the short arm, corresponding to the nucleolar organizer region (Ag-NOR), similar to the erethizontid *Sphiggurus villosus*, 2n = 42 and FN = 76, and different from the echimyids, in which the secondary constriction is interstitial.

**Conclusion:**

Both molecular phylogenies and karyotypical evidence indicated that *Chaetomys *is closely related to the Erethizontidae rather than to the Echimyidae, although in a basal position relative to the rest of the Erethizontidae. The high levels of molecular and morphological divergence suggest that *Chaetomys *belongs to an early radiation of the Erethizontidae that may have occurred in the Early Miocene, and should be assigned to its own subfamily, the Chaetomyinae.

## Background

The family Erethizontidae, the New World porcupines, is widely considered a primitive clade among caviomorph rodents, and probably diverged early in the evolutionary history of the New World hystricognaths (e.g. [1-3]). Some authors have suggested that the family may represent an independent early invasion of hystricognath rodents in South America (e.g. [1]), as the family Hystricidae may represent a separate colonization of hystricognaths in Africa [4]. The Erethizontidae is restricted to the New World and comprises about 15 extant species [5].

In a study on Neotropical porcupines, Voss and Angermann [6] clarified the taxonomy of some erethizontids. However, Bonvicino et al. [7] noted that the status of several taxa in this family and their phylogenetic relationships are still poorly understood. *Erethizon *and *Echinoprocta *are recognized as monotypic genera, whereas other species of erethizontids are allocated either to the genera *Coendou *and *Sphiggurus *(e.g [8,9]) or solely to the genus *Coendou *(e.g. [6,10]). Bonvicino et al. [7] used the mitochondrial cytochrome *b *gene and karyologic data to clarify the taxonomic status of *Coendou *and *Sphiggurus*. Both kinds of data demonstrated that *Coendou *and *Sphiggurus *represent two evolutionary lineages. Their comparative analyses of the karyotypes showed that species of *Coendou *are karyologically conservative, sharing the same diploid and fundamental numbers. Species of *Sphiggurus*, on the other hand, diverge in diploid number although they share the same fundamental number.

There are countless taxonomic issues involving the Erethizontidae, but perhaps no taxon has aroused more controversy than the genus *Chaetomys*, which contains a single species, the thin-spined porcupine *Chaetomys subspinosus*. This species is endemic to the Atlantic Rainforest in eastern Brazil and, according to Woods and Kilpatrick [9], it is found from the southern part of the state of Sergipe to the northern part of the state of Rio de Janeiro, including easternmost Minas Gerais. *Chaetomys subspinosus *is considered an endangered species by the U.S. Endangered Species Act, U.S. ESA; a vulnerable species by the International Union for the Conservation of Nature and Natural Resources, IUCN; and a threatened species by the Instituto Brasileiro do Meio Ambiente e dos Recursos Naturais Renováveis, IBAMA.

In this species, the structure of the feet, nose, and tail resembles that of the erethizontids, although there is no consensus as to whether the tail is prehensile [5,11] or not [8]. The structure of the cheek teeth, nevertheless, differs from that of the erethizontids. Based on tooth structure, Stehlin and Schaub [12] included *Chaetomys *in the family Echimyidae. Again emphasizing the molar tooth structure, Schaub [13] later assigned *Chaetomys *to the echimyid subfamily Echimyinae. Patterson and Wood [4] reasoned that two characters are fundamental to the familial assignment of *Chaetomys*. Both of these characters are strongly negative as regards erethizontid affinities, and one is strongly positive as regards echimyid affinities: (1) in contrast to the known erethizontids and in agreement with all other living caviomorphs, *Chaetomys *lacks a posterior carotid foramen; (2) in agreement with all echimyids and in contrast to all other caviomorphs, the deciduous premolars (dP4) are retained throughout life in *Chaetomys*. Patterson and Wood [4] suggested classifying *Chaetomys *in a subfamily of the Echimyidae, the Chaetomyinae, and were followed by others (e.g. [8,14]). Woods [14], for instance, divided the family Echimyidae into five subfamilies: Chaetomyinae, Dactylomyinae, Echimyinae, Eumysopinae, and the extinct Heteropsomyinae.

The placement of *Chaetomys *within the echimyids was questioned by Martin [15] who argued that *Chaetomys *lacks a derived incisor enamel microstructure, characteristic of the superfamily Octodontoidea, which includes the Echimyidae. Martin [15] also found that the posterior carotid foramen is actually present in *Chaetomys*, refuting claims by Patterson and Wood [4]. Martin [15] noted, however, that the presence of a posterior carotid foramen and the primitive incisor enamel microstructure should be regarded as plesiomorphic traits for the Hystricognathi.

Although Martin [15] found no evidence against the retention of the dP4 in *Chaetomys*, such evidence was later found [16]. Nevertheless, the substitution of the dP4 is considered a plesiomorphic trait for the Hystricognathi and again supports the exclusion of *Chaetomys *from the family Echimyidae, but does not add information on further taxonomic affinities. Carvalho [16] noted, however, that according to Bryant and McKenna [2], the presence of an internal carotid artery, although a primitive character for the Rodentia, emerges as derived character for the Erethizontidae within the hystricognaths. Carvalho [16] therefore reinterpreted the presence of the posterior carotid foramen as evidence for the association of *Chaetomys *with the erethizontids.

While the familial classification of *Chaetomys *seems to be resolved, its association with the other erethizontids is still unclear. Some authors consider its unique morphology as evidence of its distance from the rest of the Erethizontidae and classify *Chaetomys *in a separate subfamily (e.g. [9,17]). Nevertheless, *Chaetomys *and the other South American porcupines share a highly derived morphology of the hind foot that is not seen in the North American form.

In the present study, we reconstructed phylogenies based on the mitochondrial cytochrome *b *gene sequences from a single specimen of *Chaetomys subspinosus *collected in Salvador, state of Bahia, Brazil, and from representatives of seven hystricognath families: the caviomorphs Erethizontidae, Echimyidae, Ctenomyidae, Caviidae, and Octodontidae; and the phiomorphs Hystricidae and Bathyergidae. We also compared the karyotype of this specimen with those of other hystricomorphs. Our main goal was to discuss the taxonomic affinities of *C. subspinosus *on the grounds of a phylogenetic analysis.

## Results

### Karyotype

**Figure 1:**
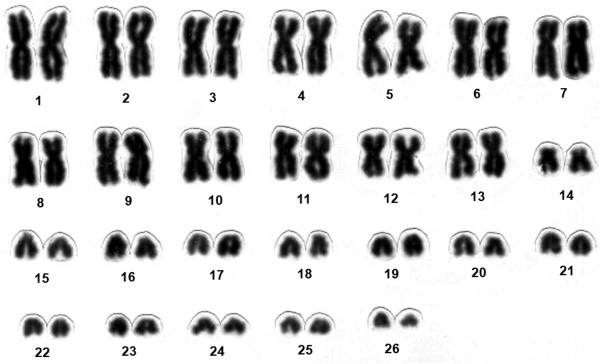
**Conventionally stained karyotype of a female of *Chaetomys subspinosus***. 2n = 52 and FN = 76; assuming that the X chromosome is biarmed; the sexual pair could not be identified.

**Figure 2:**
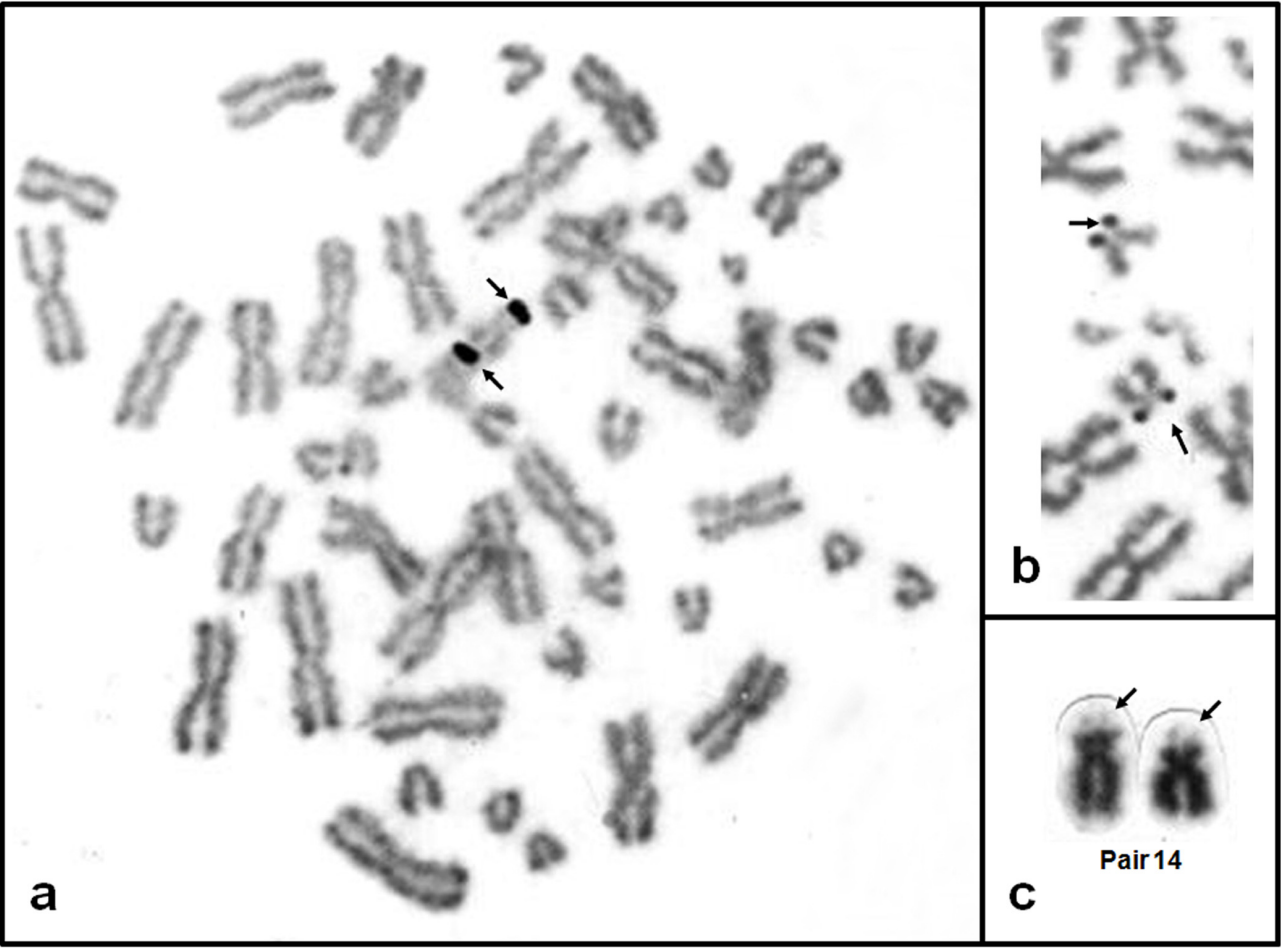
**Silver-nitrate stained NOR (Ag-NOR) metaphases of *Chaetomys subspinosus***. Complete (a) and partial (b) Ag-NOR metaphases of *Chaetomys subspinosus *showing signals on the short arm of pair 14 (arrows). (c) Conventionally stained pair 14 showing terminal secondary constriction on the short arm (arrows).

**Figure 3:**
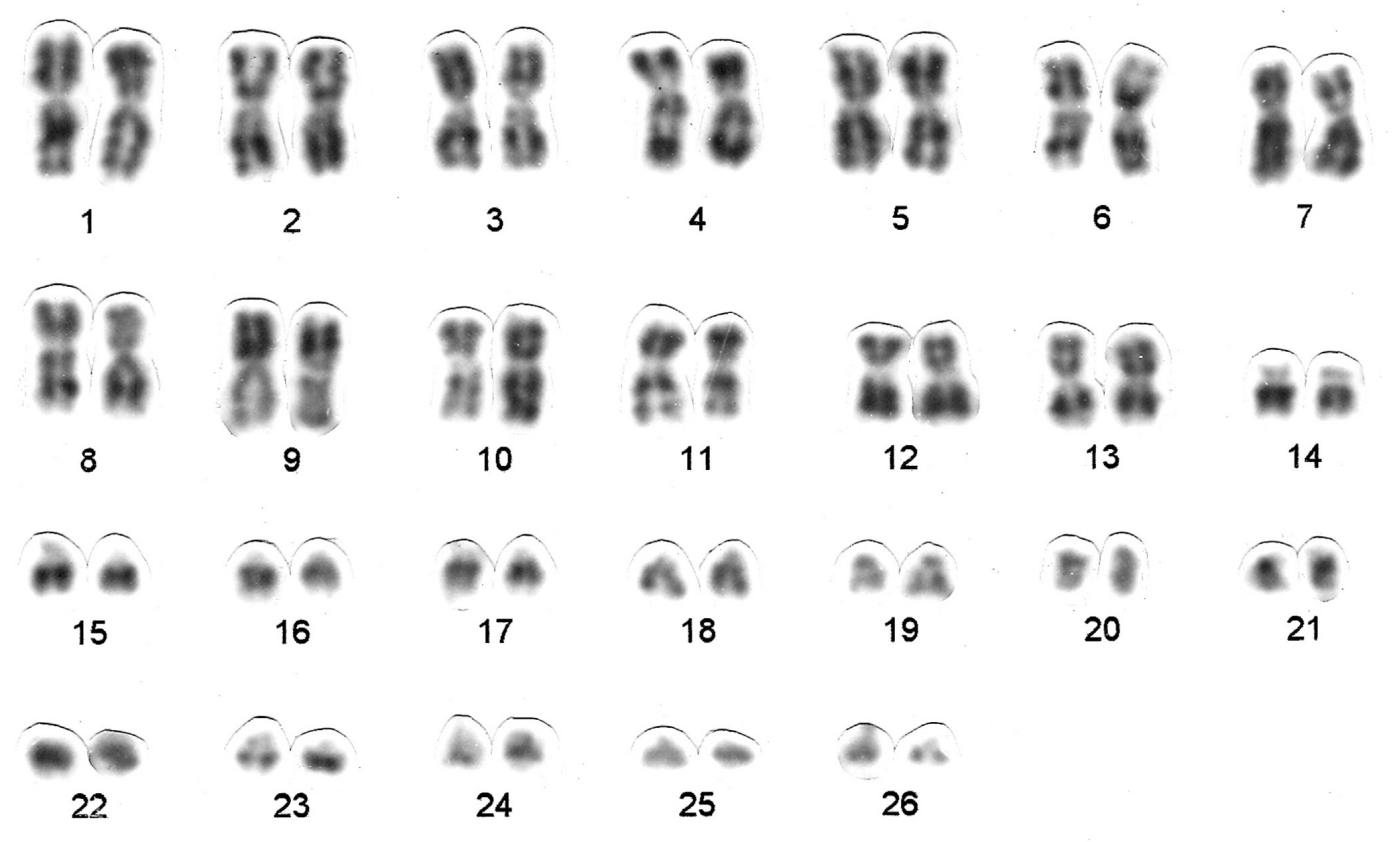
**G-banded karyotype of *Chaetomys subspinosus***.

The conventionally stained karyotype of one female of *Chaetomys subspinosus *had 2n = 52 (Figure 1). The karyotype included 13 large to medium pairs of metacentric and submetacentric chromosomes, gradually decreasing in size (pairs 1 to 13); one small pair of subtelocentric chromosomes (pair 14); and 12 small pairs of acrocentric chromosomes (pairs 15 to 26). Although the sexual pair could not be identified, we assumed that the X chromosome is biarmed, considering that: (1) the X chromosome of most placental mammals comprises about five percent of the genome; (2) the X chromosome in hystricognaths is rarely small and often biarmed; (3) all the acrocentric chromosomes of *Chaetomys subspinosus *are small. We therefore calculated the fundamental number, i.e. the number of autosome arms, as FN = 76. There was a secondary constriction, terminal to the short arm of the subtelocentric pair 14 (Figure 2c), corresponding to the nucleolar organizer region, Ag-NOR (Figures 2a and 2b). The G-banding pattern allowed us to pair homologues (Figure 3).

### Base composition and sequence variation

Polymerase chain reaction amplifications yielded, with one exception (see Methods), single products of the expected sizes. Alignment of the cytochrome *b *gene sequences of 27 taxa resulted in 1,140 base pairs, corresponding to 379 amino acids and a stop codon. Translation of the nucleotide sequences found no unexpected intermediate stop codon. The dataset contained 526 constant sites, and 89 variable characters were parsimony-uninformative. The possibility for evolution at the nucleotide level varied among codon positions. Of 525 parsimony-informative sites, 135 were at first positions, 46 at second positions, and 344 at third positions. The empirically observed ratio of rate of change among codon positions was 3:1:9.

The mean base compositions across all taxa were T = 29.9%, C = 26.9%, A = 30.9%, and G = 12.4%. Although there was a deficit of guanine, its frequency differed drastically among the three codon positions, representing 3.2% of the third positions, 13.6% of the second positions, and 20.5% of the first positions. The first and third positions were richer in adenine (30.1% and 41.8%, respectively), and the second positions had more thymine (41.1%). These frequencies reflected the strongly biased base composition and codon usage found in cytochrome *b*, and agreed with previous findings [18-20].

The g1 statistic, used to examine 1,000,000 randomly generated topologies (mean length = 4,170 steps, SD = 76.09, and g1 = -0.71), indicated the strong phylogenetic signal conveyed by this data set. In Xia's test for substitutions saturation [21] the critical index of substitution saturation depends on the topology. We found little saturation for any topology in the second positions; whereas we found little saturation for symmetrical trees and substantial saturation for asymmetrical trees in the first and third positions. The plot of transition and transversion rates at each codon position against the Kimura's 2-parameter distances for pairwise comparisons of cytochrome *b *gene sequences of our sample showed evidence for substantial saturation only at third-position transitions, and evidence for moderate saturation at third-position transversions and first-position transitions. In a previous study involving cytochrome *b *gene analysis of representatives of 11 sciurognath and 3 hystricognath families, Montgelard et al. [22] observed that homoplastic saturation events occur in some transversions along with transitions. More surprisingly, they observed that A-G transitions at third positions are less affected by saturation, showing that transitions in third positions may carry phylogenetic information.

### Phylogenetic analyses

All topologies recovered in our analyses strongly supported *Chaetomys subspinosus *as a sister clade to the erethizontids. In Table 1 we summarize the optimal branch lengths and support values for the principal nodes in our phylogenies. Maximum parsimony (MP) heuristic search produced 182,821,196 rearrangements, and resulted in one most-parsimonious tree (3,144 steps, CI = 0.33, RI = 0.44). Hierarchical likelihood ratio tests (hLRTs) as well as the Akaike information criterion (AIC) selected as the best-fit model for our dataset the general-time reversible model with a proportion of invariable sites and a discrete gamma distribution for the variable sites (GTR+I+Γ) (lnL = -13,066.6846). The estimated gamma shape parameter (α) was 0.5884 and the proportion of invariable sites was 0.3962. Maximum likelihood (ML) heuristic search produced 191,071 rearrangements, and resulted in one best tree (-lnL 13,055.69138). The majority-rule consensus of 59,900 sampled trees reconstructed from two runs of Bayesian analysis (BA) generated a topology similar to the ML best tree. For the estimated marginal likelihoods in BA, the arithmetic mean was -13,080.24, and the harmonic mean was -13,113.13.

**Table 1:** Lengths of optimal branches and robustness estimators for representative nodes of the Hystricognathi cytochrome *b *trees.

**Nodes**	**Inference methods**							
	
	**MP**				**ML**		**BA**	
			
	**steps**	**BP**	**steps**	**DI**	**steps**	**BP**	**steps**	**BP**
Hystricognathi	86	**100**	83	**33**	85	**100**	87	**100**
Bathyergidae	60	**100**	60	**20**	58	**100**	58	**100**
Hystricidae + Caviomorpha	46	72	42	5	48	82	45	**98**
Caviomorpha	58	50	*	*	55	67	55	**100**
Hystricidae + Erethizontidae	*	38*	52	3	*	*	*	*
Erethizontidae	49	**97**	70	**17**	54	**100**	51	**100**
Erethizontinae	59	**100**	68	**22**	58	**99**	59	**100**
*Coendou *+ *Sphiggurus*	43	**92**	44	7	43	**95**	43	**98**
*Coendou*	52	**100**	52	**36**	52	**100**	52	**100**
*Sphiggurus*	60	**100**	60	**43**	64	**100**	60	**100**
Octodontoidea + Cavioidea	51	59	36	5	52	71	49	**100**
Octodontoidea	72	**96**	49	**14**	56	**99**	51	**100**
Echimyidae	*	42*	34	5	38	**91**	37	**100**
Octodontidae + Ctenomyidae	*	41*	40	5	38	79	45	93
Ctenomyidae	115	**100**	70	**37**	76	**100**	77	**100**
Octodontidae	97	**96**	49	11	48	**100**	47	**100**
Cavioidea	55	66	54	6	49	**92**	51	**100**
Caviidae	40	63	39	6	48	75	42	84

Lengths of optimal internal branches are given in number of steps. Bootstrap percentages (BP) were obtained using phylogenetic reconstructions under maximum parsimony (MP) and maximum likelihood (ML). Bremer support or decay index (DI) was implemented in MP. Support for the Bayesian analysis (BA) was given by Bayesian posterior probabilities (BPP). BP and BPP were estimated under 50% majority-rule consensus. Star (*) indicates that the node was not recovered or was not supported in the corresponding analysis. Support values within Zander's [50] 0.95 binomial confidence intervals (CI) are highlighted in bold face.

The permutation tail probability (T-PTP) test [23] supported the inclusion of *Chaetomys *within the family Erethizontidae (P = 0.000270) against its inclusion within the family Echimyidae (P = 0.875800). The Templeton [24] test found no difference between the best unconstrained tree and the best constrained tree to include *Chaetomys *within the Erethizontidae, whereas it found a significant difference (p < 0.001) between the best unconstrained tree and the best tree constrained to include *Chaetomys *within the Echimyidae. The Kishino-Hasegawa (KH) [25] and Shimodaira-Hasegawa (SH) [26] tests gave similar results.

### Molecular evolutionary rates and molecular dating

**Figure 4:**
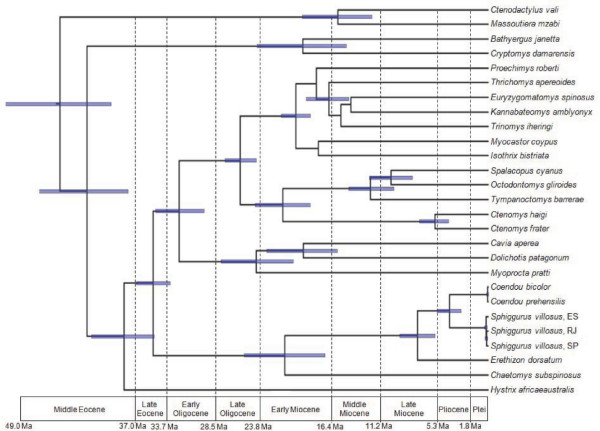
**Divergence time estimates from the Bayesian analyses (BA), of cytochrome *b *sequences, of 25 hystricognaths and the outgroup**. Molecular time-scale for the Hystricomorpha. The chronogram was obtained using the Maximum Clade Credibility Tree (MCC) of phylogenetic reconstructions sampled under Bayesian Markov chain Monte Carlo (MCMC) method, with rates conformed to a molecular clock (CLOC). The divergence times correspond to the mean posterior estimate of their age in millions of years (Ma). The blue bars represent the 95% HPD interval for the divergence time estimates. The geological epochs are reported according to the 1999 Geologic Time Scale of the Geological Society of America (Plei = Pleistocene). ES, Espírito Santo; RJ, Rio de Janeiro; SP, São Paulo.

The likelihood ratio test (LRT), comparing likelihood scores of unconstrained and clock-constrained best trees, was not significant at the alpha level of 0.0100 (lnL = -13,076.26581 under global clock constraint versus lnL = -13055.69138 without clock constraint; LRT statistics = 41.148438, df = 25, *P *= 0.022146), suggesting clock-like behavior. The estimates of divergence times calculated using Bayesian analyses are shown in Table 2, and the chronogram constructed using Bayesian analysis assuming rates conformed to a molecular clock is shown in Figure 4. The estimates of divergence times calculated using non-Bayesian methods are shown in Table 3. The results from Bayesian and non-Bayesian methods were slightly different; the greatest discrepancy was found in the deeper nodes, namely the Hystricomorpha and the Ctenodactylidae. The NPRS-LOG method yielded the most divergent ages, and the GRMD yielded intermediate ages. As expected, the methods assuming relaxed rates (UCLN and NPRS-LOG) had greater variance of ages than the methods assuming clock-like rates (CLOC and GRMD). For most nodes, nevertheless, the estimates of divergence times using different methods were similar, within the same or nearly the same geological epochs.

**Table 2:** Estimates of mean divergence times, and respective 95% HPD intervals given by Bayesian analyses of cytochrome *b *nucleotide sequences.

**Nodes**	**CLOC**		**UCLN**		**Geological epoch**
			
	**Mean**	**95% HPD**	**Mean**	**95% HPD**	
Hystricomorpha	44.5	39.2–50.1	47.1	39.8–55.3	Middle Eocene
Ctenodactylidae	15.6	12.2–19.3	16.1	10.0–22.2	Middle Miocene
Hystricognathi	41.7	37.4–46.6	43.7	37.9–50.1	Middle Eocene
Bathyergidae	19.3	14.8–24.0	20.1	12.8–27.7	Early Miocene
Caviomorpha	34.8	33.1–36.6	34.8	33.0–36.5	Late Eocene
Erethizontidae	21.2	17.0–25.4	21.0	15.1–27.0	Early Miocene
Erethizontinae	7.4	5.6–9.2	7.7	5.1–10.4	Late Miocene
*Coendou *+ *Sphiggurus*	4.1	2.9–5.4	4.2	2.5–6.0	Early Pliocene
*Coendou*	0.1	0.04–0.2	0.1	0.04–0.2	Pleistocene
*Sphiggurus*	0.3	0.2–0.5	0.3	0.2–0.5	Pleistocene
Octodontoidea	25.8	24.1–27.3	25.8	24.2–27.5	Late Oligocene
Echimyidae	20.0	18.5–21.5	20.0	18.4–21.7	Early Miocene
Ctenomyidae	5.6	4.2–7.2	5.6	3.4–8.1	Late Miocene
Octodontidae	12.3	9.8–14.5	12.5	9.2–16.1	Middle Miocene
Cavioidea	24.1	20.3–27.8	23.1	18.1–28.0	Late Oli.-Early Mio.
Caviidae	19.5	15.5–23.7	19.1	13.6–25.1	Early Miocene

Estimates of divergence times expressed in million years. CLOC: rates conformed to a molecular clock. UCLN: rates uncorrelated, with the rate in each branch independently drawn from a lognormal distribution. HPD: highest posterior densities. The geological epochs corresponding to divergence times followed the 1999 Geologic Time Scale of the Geological Society of America. Oli.: Oligocene; Mio.: Miocene.

**Table 3:** Estimates of mean divergence times, and respective 95% CL intervals given by non-Bayesian analyses of cytochrome *b *nucleotide sequences.

**Nodes**	**NPRS-LOG**		**GRMD**		**Geological epoch**
			
	**Mean**	**95% CL**	**Mean**	**95% CL**	
Hystricomorpha	58.6	53.7–76.1	52.2	49.1–63.1	Late Pal.-Early Eoc.
Ctenodactylidae	26.3	22.7–33.4	17.68	14.8–20.5	Late Oli.-Early Mio.
Hystricognathi	46.4	42.4–54.0	44.1	41.5–49.6	Middle Eocene
Bathyergidae	23.5	17.8–26.9	20.9	16.6–24.2	Early Miocene
Caviomorpha	34.0	-	34.0	-	Late Eocene
Erethizontidae	23.9	22.3–26.4	22.2	18.9–25.9	Late Oli.-Early Mio.
Erethizontinae	9.5	7.2–14.8	7.7	3.4–9.5	Late Miocene
*Coendou *+ *Sphiggurus*	5.1	2.7–10.7	4.5	2.6–5.5	Late Mio.-Early Pli.
*Coendou*	0.1	0.1–0.7	0.1	0.02–0.2	Pleistocene
*Sphiggurus*	0.2	0.1–0.8	0.2	0.1–0.5	Pleistocene
Octodontoidea	27.0	-	27.0	-	Late Oligocene
Echimyidae	20.0	-	20.0	-	Early Miocene
Ctenomyidae	5.9	3.9–8.7	5.4	3.7–6.5	Late Miocene
Octodontidae	15.2	10.2–17.9	13.1	10.2–15.1	Middle Miocene
OGL+SCY	12.1	7.0–15.4	10.6	7.4–12.2	Middle-Late Miocene
Cavioidea	25.9	16.7–28.1	23.7	16.1–26.1	Late Oli.-Early Mio.
Caviidae	22.0	16.0–25.7	19.7	15.5–23.3	Early Miocene

Estimates of divergence times expressed in million years. NPRS-LOG: nonparametric rate smoothing with log-scale rates. GRMD: global rate minimum deformation method. CL: confidence limits. The geological epochs corresponding to divergence times followed the 1999 Geologic Time Scale of the Geological Society of America. Pal.: Paleocene; Eoc.: Eocene; Oli.: Oligocene; Mio.: Miocene; Pli.: Pliocene.

## Discussion

### A species-specific karyotype

The karyotype observed in *Chaetomys subspinosus *differs in diploid (2n = 52) and fundamental (FN = 76) numbers from all echimyid or erethizontid karyotypes investigated so far, suggesting that this karyotype is species-specific for *Chaetomys subspinosus*.

Echimyids have diploid numbers ranging from 2n = 14–16 in *Proechimys *gr. *goeldii *[27] to 2n = 118 in *Dactylomys boliviensis *[28], the latter being the largest diploid number described for a mammal. The Echimyidae is therefore the family with the widest diversity in diploid numbers found in mammals. Of all the echimyid species studied to date, only two have 2n = 52: *Proechimys guairae*, with FN = 72–74 [29]; and *Phyllomys nigrispinus*, with FN = 94 [30]. Both karyotypes differ in chromosome morphology from that of *Chaetomys subspinosus*. Erethizontids have diploid numbers ranging from 2n = 42 in *Erethizon dorsatum *[31], *Sphiggurus pruinosus *[32], and *S. villosus *[33] to 2n = 74 in all species of *Coendou *studied so far [34,35], and none of them has 2n = 52.

Karyotypes with fundamental numbers of 76 are found in only two echimyid species: *Proechimys urichi*, with 2n = 62 [36]; and *Proechimys poliopus*, with 2n = 42 [36]. Chromosome morphology in both species differs from that in *Chaetomys subspinosus*. The fundamental numbers in erethizontids are FN = 76 in *Erethizon dorsatum *[31] and all species of *Sphiggurus *studied so far [7,32,33,35], and FN = 82 in all species of *Coendou *studied so far [34,35].

Although different, the diploid number of *Chaetomys subspinosus *is intermediate relative to the erethizontids, and the fundamental number is the same as that found in *Erethizon *and *Sphiggurus*. The FN shared by these genera suggests that their karyotypes can be derived from one another by Robertsonian rearrangements, and that the ancestral erethizontid karyotype may have had a fundamental number of FN = 76.

**Figure 5:**
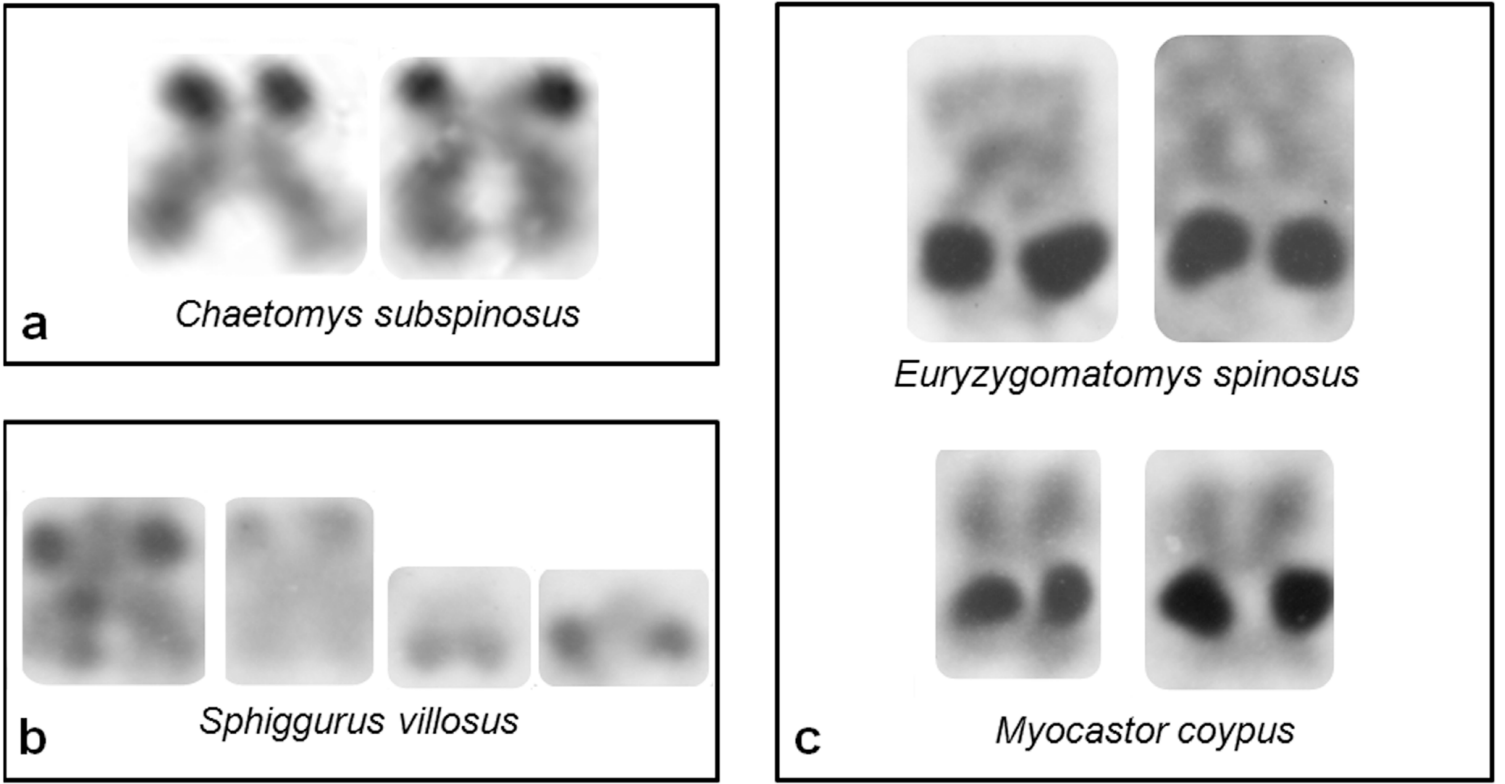
**NOR-bearing chromosomes found in Chaetomys subspinosus, Sphiggurus villosus, Euryzygomatomys spinosus, and Myocastor coypus**. Comparison of NOR positions in chromosomes of: (a) *Chaetomys subspinosus*, (b) the erethizontid *Sphiggurus villosus*, and (c) the echimyids *Euryzygomatomys spinosus *and *Myocastor coypus*.

The Ag-NOR-bearing pair found in *Chaetomys subspinosus *resembles one of two Ag-NOR pairs found in the erethizontid *Sphiggurus villosus *(Figure 5), and differs from the Ag-NOR pair found in echimyids. In *Chaetomys*, as well as in *Sphiggurus*, there was a secondary constriction, associated with the Ag-NOR, terminal to the short arm of a subtelomeric pair. There is a secondary constriction associated with the Ag-NOR, in a single chromosome pair, in all echimyid species studied so far; however, it is in a metacentric pair and is interstitially located in the long arm.

Leal-Mesquita [37] observed that G-banding patterns flanking the Ag-NORs are rather conservative across different echimyid species. Interstitial Ag-NORs, similar to that found in echimyids, are also found in a few insectivores, cetartiodactyls, chiropterans, and primates, and frequently among carnivores [38-40]. In this last group, the pair with interstitial Ag-NORs is referred to as the 'carnivore chromosomes'. Among rodents, all ctenodactylids and most hystricognaths have a single pair of chromosomes with interstitial Ag-NORs, e.g., thryonomids, some hystricids, hydrochaerids, cuniculids, chinchillids, and octodontoids [34,39]. Even the tetraploid octodontid *Tympanoctomys barrerae *has a single pair of active NORs, although signals of the presence of rDNA clusters were detected in four chromosomes with in-situ hybridization using rDNA probes [41].

**Figure 6:**
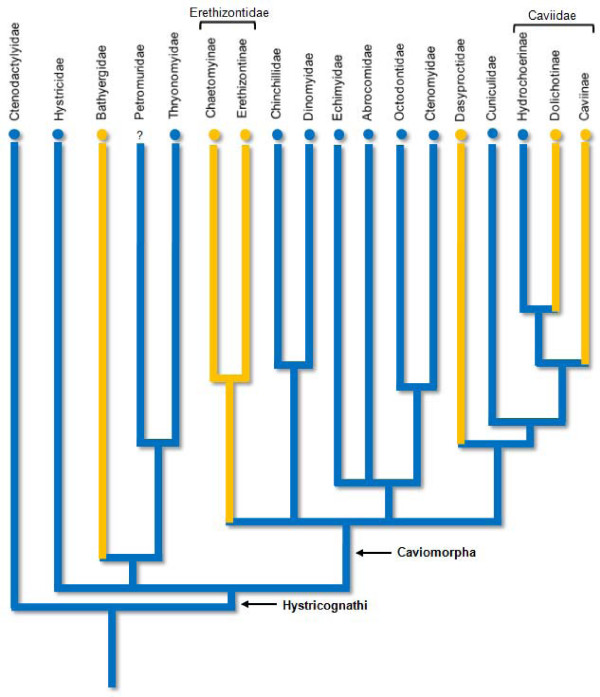
**Phylogeny of the Hystricomorpha**. Distribution of the single pair of interstitial-NOR-bearing chromosomes as a character in the phylogeny of Hystricomorpha based on published data [43,45-47,51,56-58,81-84] and data herein presented. Blue branches indicate lineages with interstitial NORs. Yellow branches indicate lineages with terminal NORs. Blue circles indicate taxa with one pair bearing interstitial NORs. Yellow circles indicate taxa with one or more pairs bearing terminal NORs.

Although the interstitial-Ag-NOR pair is absent in bathyergids, erethizontoids, dasyproctids, and caviids [39]; it appears to be a plesiomorphic character for the hystricomorphs. By plotting the interstitial-Ag-NOR pair condition as a plesiomorphic character at the root of a phylogeny of the diversification of Hystricomorpha, as considered by Brandt [42], based on published information and our data, we have found that fewer changes are needed to reach the present pattern of Ag-NOR distribution in hystricomorphs, than by plotting the interstitial-Ag-NOR pair condition as a derived character (Figure 6). Actually only five Ag-NOR position changes, against seven in the competing hypothesis, would be necessary to form the present distribution pattern of this character: one in the Bathyergidae, one in the Erethizontidae, one in the Dasyproctidae, and two in the Caviidae.

### Sister-group to the erethizontids

In South America, rodents derive from two main distinct colonizations: the hystricognath, which is well represented from the end of Eocene to the present; and the sciurognath (Muroidea, Sciuroidea and Geomyoidea), which entered the continent later, in a series of invasions at the end of the Miocene that intensified during the Pliocene [11]. The hystricognaths are traditionally divided into two groups: the Old World hystricognaths, the Phiomorpha; and the New World hystricognaths, the Caviomorpha. Although the monophyly of the Hystricognathi seems to be well resolved, this is not the case for the Phiomorpha and Caviomorpha. Woods [14], for example, considered the term "Caviomorpha" inappropriate, because it is unlikely that the New World hystricognaths derived from a single radiation, and suggested that it is best to discuss them in their superfamilies: Erethizontoidea, Chinchilloidea, Cavioidea, and Octodontoidea.

Several authors have reached conflicting conclusions: some found support for the monophyly of Phiomorpha and Caviomorpha (e.g. [43,44]); others found support for the monophyly of Caviomorpha but not for Phiomorpha (e.g. [45-47]); and still others found no support for the monophyly of either of them (e.g. [48,49]).

In our analyses, the Hystricognathi formed a monophyletic group with 100% support in all topologies and a decay index (DI) 33 in MP. According to Zander [50], the minimum indexes necessary for a binomial confidence interval (CI) of 0.95 for branch lengths of about 60 steps are 88% for bootstraps (BP), 91% for Bayesian posterior probabilities (BPP), and 15 for DI. We thus considered the monophyly of Hystricognathi to be strongly corroborated in all topologies.

The Phiomorpha, including the family Hystricidae, was nonmonophyletic in any of our phylogenies. Instead, we recovered a monophyletic group joining Hystricidae and the caviomorphs with moderate support, except for the BA in which support was strong. The Caviomorpha was monophyletic in ML and BA, but not in MP. In the most-parsimonious tree, the phiomorph family Hystricidae was a sister-group to the caviomorph family Erethizontidae, although this relationship was poorly supported. In contrast, in the MP bootstrap and in the ML and BA analyses, the Hystricidae was sister-group to a monophyletic Caviomorpha, although this relationship was strongly supported only by the BPP. The most-parsimonious-tree topology agreed with authors who have advocated against a single colonization event of South America by hystricognaths (e.g. [2,17]), whereas the ML and BA topologies agreed with authors who have advocated for a single colonization event (e.g. [51,52]).

The superfamily Octodontoidea was monophyletic and strongly supported by all estimators; in fact, this group is well supported by a number of studies (e.g. [43,46,47]). The monophyly of the family Octodontidae was strongly supported by the BPs and the BPP, but only moderately supported by the DI. The monophyly of the family Ctenomyidae was strongly supported by all estimators. The association of Ctenomyidae with Octodontidae, although recovered in all topologies, had little support in all estimators. Thus, the inclusion of *Ctenomys *to the Octodontidae was not supported.

Although the monophyly of the family Echimyidae was recovered in all topologies, it was strongly supported only in the ML and BA analyses. Relationships between echimyid genera were poorly resolved, similarly to previous studies using the cytochrome *b *gene [20,53].

The superfamilies Octodontoidea and Cavioidea appeared as sister-clades in all topologies, but this was strongly supported only by the BPP. The monophyly of Cavioidea had strong support in the ML and BA analyses, whereas the monophyly of Caviidae, although it was recovered in all topologies, had little support.

The genera *Coendou *and *Sphiggurus *formed a monophyletic group, well supported by all indicators except the DI. In all trees, *Coendou *was monophyletic with strong support, as was *Sphiggurus*. *Erethizon *was sister-clade to the group formed by *Coendou *and *Sphiggurus*, with strong support in all topologies.

The phylogenetic reconstructions recovered *Chaetomys *as a sister-clade to the erethizontids, with strong support, in all topologies by all estimators. Furthermore, all the statistical hypothesis tests (T-PTP, Templeton, KH, and SH tests) supported the monophyly of *Chaetomys *with the erethizontids, whereas none of them supported the monophyly of *Chaetomys *with the echimyids. These findings support the inclusion of *Chaetomys *within the family Erethizontidae, as proposed by Martin [15] and Carvalho [16]. The basalmost position of *Chaetomys *within the Erethizontidae suggests that the highly derived morphology of the hind foot shared by *Chaetomys *and the other South American porcupines is a convergent character.

### Two subfamilies

The mean of the genetic ML-corrected distances between the *Chaetomys *haplotype and the erethizontids was 20.2% (SD = 0.6%). This value contrasts with the mean of the ML distances between *Chaetomys *and the echimyids, 24.7% (SD = 0.5%), but is similar to the distances between Echimyidae and Ctenomyidae, 20.0% (SD = 0.6%); Echimyidae and Octodontidae, 19.2% (SD = 0.9%); and Ctenomyidae and Octodontidae, 19.1% (SD = 0.6%). However, although the mean of the ML distances between echimyid haplotyopes was 17.9% (SD = 1.0%), the greatest distance between two echimyids was 20.1%, between *Myocastor coypus *and *Trinomys iheringi*.

The level of morphological and molecular divergence between *Chaetomys *and the other erethizontids, supports the inclusion of *Chaetomys *in its own subfamily within the family Erethizontidae. We shall therefore refer to *Chaetomys *as Chaetomyinae and to the other erethizontids as Erethizontinae.

The distances of Chaetomyinae and Erethizontinae differed considerably when compared with other taxa. Between Chaetomyinae and other caviomorph families, the means of ML distances ranged from 24.4 to 26.2%; and between Erethizontinae and other caviomorphs, the means of ML distances ranged from 22.3 to 23.1%. Between Chaetomyinae and Hystricidae, the ML distance was 22.4%; and between Erethizontinae and Hystricidae, the mean of ML distances was 21.4% (SD = 0.4%). Only between Chaetomyinae and Bathyergidae, the mean of ML distances, 24.8% (SD = 0.5%), was similar to the mean of ML distances between Erethizontinae and Bathyergidae, 24.5% (SD = 0.4%). Finally, the mean of ML distances between Chaetomyinae and Ctenodactylidae was 26.1% (SD = 0.1%); whereas between Erethizontinae and Ctenodactylidae it was 24.7% (SD = 0.4%).

The levels of divergence between *Chaetomys *and the other taxa in the sample were rather high compared to its sister-clade, the Erethizontinae. If we assume that divergence times between two lineages and their outgroup are the same, any discrepancy in the branch lengths should be ascribed to differences in substitution rates. Our data suggest, therefore, that the higher divergence levels in *Chaetomys *may be the result of higher evolutionary rates.

One could argue that the sequence of *Chaetomys *presented herein might, in fact, be an inactive copy of the mitochondrial cytochrome *b *gene, that is, a pseudogene. This could explain the high substitution rates, because of a lack of functional constraints. Nevertheless the base frequencies in the sequence of *Chaetomys *(T = 30.4%, C = 27.8%, A = 30.6%, G = 11.2%) are similar to the mean of the frequencies of the entire sample, although with fewer Gs than any other sequence in the sample. In the third-codon position the frequency of Gs is 2.1%, a bias commonly found in the cytochrome *b *of other rodents [18,19], which contrasts with the frequencies of Gs in the first and second positions, 18.9% and 12.6%, respectively. Furthermore, the amino-acid sequence resulting from the translation of the nucleotide sequence of *Chaetomys*, did not contain any anomalous premature stop codon or changes in the reading frame.

The mean of ML distances between *Erethizon dorsatum *and species of *Coendou *and *Sphiggurus *was 13.7% (SD = 0.4%). This is less than the smallest distance between two octodontids, 14.4% between *Octodontomys gliroides *and *Spalacopus cyanus*. The mean of ML distances between species of *Coendou *and *Sphiggurus *was 11.2% (SD = 0.1%). These distances are at the same level as the distance between the two species of *Ctenomys*, 11.1%; less than the smallest distance between two echimyids, 15.8% between *Kannabateomys amblyonyx *and *Euryzygomatomys spinosus*; and less than the distances between caviid, bathyergid, or ctenodactylid genera, 19.2, 18.8, or 16.7%, respectively. Even though we found strong support for a reciprocal monophyly between *Coendou *and *Sphiggurus*, although the monophyly of *Coendou *and *Sphiggurus *as a group was strongly supported by all estimators but the DI, the levels of divergence do not support the separation of these genera. A larger sample, including *Echinoprocta *and more species of *Coendou *and *Sphiggurus*, will be necessary to clarify this matter.

The origin of hystricognaths dating from the Middle Eocene is consistent with most previous studies [43,46,54,55]. Our estimates for the origin of caviomorphs dated from the Late Eocene, whereas previous estimates range from the Middle Eocene [47,56], to the Late Eocene [47,55-58], or the Early Oligocene [46,59]. The separation of Echimyidae from Octodontidae and Ctenomyidae would have occurred in the Late Oligocene, in nearly the same epoch as the separation of Caviidae and Dasyproctidae, in the Late Oligocene to the Early Miocene.

The separation of Erethizontidae into Chaetomyinae and Erethizontinae would have occurred in the Late Oligocene to the Early Miocene, in nearly the same epoch as the origin of the Echimyidae and the Caviidae, in the Early Miocene. Curiously the separation of *Erethizon *from the rest of the Erethizontinae took place in the Late Miocene, which means, before the Great American Interchange that followed the formation of the Central American Landbridge, about 3.5 million years ago; indicating that its lineage may have diverged before migrating to the north. The separation of *Coendou *and *Sphiggurus *would have occurred in the Late Miocene to the Early Pliocene.

## Conclusion

These new data from sequencing of the cytochrome *b *gene and karyotyping of a female thin-spined porcupine, *Chaetomys subspinosus*, confirm that this species does not belong to the family Echimyidae. Instead, it is related to the Erethizontidae, and belongs to a sister-clade to the other erethizontids. Nevertheless, its basalmost position relative to the Erethizontidae, its high levels of sequence divergence, and its morphological distinctiveness suggest that *Chaetomys *belongs to an early radiation of the Erethizontidae that may have occurred in the Early Miocene, from 23 to 21 million years before the present, and should be allocated to a subfamily of its own, the subfamily Chaetomyinae, sister to the subfamily Erethizontinae, which contains the other erethizontid genera.

## Methods

### Karyotypes

We karyotyped a single female of *Chaetomys subspinosus*. The specimen, which had been hit by a car, was found by Bruno Martins in an Atlantic Rainforest fragment near the campus of the Universidade Federal da Bahia (UFBA), in the city of Salvador, state of Bahia, northeastern Brazil. The specimen was identified by Prof. Pedro Luís Bernardo da Rocha (UFBA) and sent alive to our laboratory for analyses.

After in-vivo colchicine treatment, chromosome preparations were obtained from bone marrow and spleen. Chromosome staining was done using Giemsa. Estimation of the fundamental number (FN) assumed that the X chromosome is biarmed (see results). Staining of the nucleolar organizer region used the silver-nitrate (Ag-NOR) technique. G-banding was carried out following routine protocol.

### Taxon sampling, DNA extraction, amplification and sequencing

For the molecular analyses, our sample consisted of one specimen of *Chaetomys subspinosus*; two specimens of the erethizontid *Sphiggurus villosus*; one specimen of the erethizontid *Erethizon dorsatum*; one specimen of each of the following six species of echimyids: *Euryzygomatomys spinosus*, *Kannabateomys amblyonyx*, *Myocastor coypus*, *Proechimys roberti*, *Thrichomys apereoides*, and *Trinomys iheringi*; and one specimen of the caviid *Cavia aperea *(Table 4). DNA of the specimens was isolated from liver or muscle preserved in ethanol or in an ultrafreezer, using 7.5 M ammonium acetate and isopropanol, following Fetzner [60]. Two overlapping fragments of the complete mitochondrial cytochrome *b *DNA were amplified in 25 μl of polymerase chain reaction (PCR) solution, using several combinations of the primers MVZ 05, MVZ 14, MVZ 16, MVZ 51, MVZ 127, and MVZ 108 [see Additional file 1] under the following temperature regime: initial denaturation 94°C/5 min, then 39 cycles of 94°C/30 s, 48°C/45 s, 72°C/1 min, and final extension at 72°C/10 min.

**Table 4:** Specimens used in the phylogenetic analyses of cytochrome *b*, corresponding GenBank Accession Numbers, locality, geographical coordinates and reference data.

**Taxon**	**GenBank Accession Number**	**Locality**	**Lat**.	**Long**.	**Reference**
**Ctenodactylidae**					
*Ctenodactylus vali*	AJ389532	--			[22]
*Massoutiera mzabi*	AJ389533	--			[22]
**Bathyergidae**					
*Bathyergus janetta*	AF012241	De Riet, SAF	-30.1	17.4	[85]
*Cryptomys damarensis*	U87526	Okavango Delta, BOT	-19.5	23.2	[86]
**Hystricidae**					
*Hystrix africaeaustralis*	X70674	--			[19]
**Erethizontidae**					
*Chaetomys subspinosus*	EU544660	Salvador, BA	-13.0	-38.5	this study
*Coendou bicolour*	U34852	Eirunepé, Rio Juruá, AM	-6.6	-60.9	[20]
*Coendou prehensilis*	AF411581	UHE Manso, MT	-15.5	-55.8	[7]
*Ertehizon dorsatum*	FJ357428	--			this study
*Sphiggurus villosus*	EU544661	UHE Rosal, ES	-20.9	-41.7	this study
*Sphiggurus villosus*	EU544662	Biritiba Mirim, SP	-23.6	-46.0	this study
*Sphiggurus villosus*	AF411580	Sumidouro, RJ	-22.1	-42.7	[7]
**Echimyidae**					
*Euryzygomatomys spinosus*	EU544667	Biritiba Mirim, SP	-23.6	-46.0	this study
*Isothrix bistriata*	L23355	Upper Rio Urucu, AM	-4.9	-65.3	[87]
*Kannabateomys amblyonyx*	EU544665	Biritiba Mirim, SP	-23.6	-46.0	this study
*Myocastor coypus*	EU544663	Biritiba Mirim, SP	-23.6	-46.0	this study
*Proechimys roberti*	EU544666	Vila Rica, MT	-9.9	-51.2	this study
*Thrichomys apereoides*	EU544668	Januária, MG	-15.5	-44.4	this study
*Trinomys iheringi*	EU544664	Boracéia, SP	-22.2	-48.8	this study
**Octodontidae**					
*Octodontomys gliroides*	AF370706	Tilcara, Jujuy, ARG	-23.6	-65.4	[88]
*Spalacopus cyanus*	AF007061	--			[89]
*Tympanoctomys barrerae*	AF007060	--			[89]
**Ctenomyidae**					
*Ctenomys frater*	AF007045	Tarija, BOL	-21.5	-64.7	[89]
*Ctenomys haigi*	AF422920	Perito Moreno, ARG	-41.1	-71.0	[53]
**Caviidae**					
*Cavia aperea*	EU544669	Biritiba Mirim, SP	-23.6	-46.0	this study
*Dolichotis patagonum*	AY382787	Santa Cruz, ARG	-50.0	-68.5	[84]
**Dasyproctidae**					
*Myoprocta pratti*	U34850	Altamira, Rio Juruá, AM	-6.6	-68.9	[20]

**Countries**: ARG, Argentina; BOL, Bolivia; BOT, Botswana; PAR, Paraguay; PER, Peru; SAF, South Africa; VEN, Venezuela. **States in Brazil**: AC, Acre; AM, Amazonas; BA, Bahia; ES, Espírito Santo; MG, Minas Gerais; MT, Mato Grosso; RJ, Rio de Janeiro; SP, São Paulo.

After an agarose gel check, PCR products were cycle-sequenced using the ABI PRISM Big Dye Terminator v 3.0 kit (Applied Biosystems) through 25 cycles of 95°C/30 s, 50°C/15 s, 60°C/4 min. Sequencing primers were the same as were used in the PCR amplifications. After purification in 75% isopropanol, and precipitation in 70% ethanol, the cycle-sequencing products were resuspended in TSR buffer (Applied Biosystems) and run on an ABI PRISM 3700 DNA Analyzer automated sequencer (Applied Biosystems).

GenBank sequences completed our dataset, adding 14 samples from eight hystricognath families: Erethizontidae (*Coendou bicolor*, *Coendou prehensilis*, and *Sphiggurus villosus*), Echimyidae (*Isothrix bistriata*), Ctenomyidae (*Ctenomys frater *and *Ctenomys haigi*), Caviidae (*Dolichotis patagonum*), Dasyproctidae (*Myoprocta pratti*), Octodontidae (*Octodontomys gliroides*, *Spalacopus cyanus*, and *Tympanoctomys barrerae*), Hystricidae (*Hystrix africaeaustralis*), and Bathyergidae (*Bathyergus janetta *and *Cryptomys damarensis*). As the outgroup we used two sequences, also from GenBank, from ctenodactylids: *Ctenodactylus vali *and *Massoutiera mzabi*. This family is considered a sister group to the Hystricognathi [51].

The sequences included at least one sample of each extant echimyid subfamily recognized by Woods and Kilpatrick [9]: Dactylomyinae (*Kannabateomys amblyonyx*), Echimyinae (*Isothrix bistriata*), and Eumysopinae (*Euryzygomatomys spinosus*, *Proechimys roberti*, *Thrichomys apereoides*, and *Trinomys iheringi*). We also added *Myocastor coypus*, which is at times assigned to the subfamily Myocastorinae within the Echimyidae [17], or within the Capromyidae [61]; or to its own family, the Myocastoridae [9]. The specimens used in the present study, corresponding GenBank Accession Numbers, localities (when available) with geographical coordinates, and respective references are listed in Table 4.

### A possible pseudogene

While amplifying the mitochondrial cytochrome *b *gene, we noticed that *Chaetomys subspinosus *samples amplified using MVZ 05 and MVZ 16 primers repeatedly formed two bands rather than one, in the check gel for PCR products. One of the bands had the expected size of approximately 800 bp, whereas the second band had approximately 600 bp and was often brighter, being occasionally the only fragment to be recovered.

We concluded that this unspecific band might represent a pseudogene, that is, an inactive copy of the cytochrome *b *gene inserted within nuclear or mitochondrial genomes. Therefore we proceeded with amplification of samples from *Chaetomys subspinosus *with different primer combinations, and obtained successful results using MVZ 51 and MVZ 16 primers. The sequence obtained with these primers confirmed that the sequence of the priming site corresponding to MVZ 05 in *Chaetomys subspinosus *is different from the corresponding sequence of this primer.

### Alignment and phylogenetic analyses

For each specimen we obtained multiple strands that were assembled in the program ABI PRISM Sequence Navigator version 1.0.1 (Applied Biosystems). Alignment was performed using the program Clustal X version 1.83 [62] with default parameters.

Amino-acid translation was done through the program MacClade 4.08 [63], to verify the quality of sequences, confirming the correct reading frame positions, and finding unexpected stop codons. We used the program MEGA version 4.0 [64] to obtain estimates of nucleotide composition, nucleotide pair frequencies, and codon usage. To test for the presence of saturation, we produced, for each codon position, plots of transitions and transversions versus Kimura's 2-parameter pairwise distances; and implemented the test by Xia et al. [21]. Both methods were performed in the program DAMBE version 5.0.23 [65].

Phylogenetic reconstructions using maximum parsimony (MP) and maximum likelihood (ML) as optimality criteria were carried out with PAUP* version 4.0b10 [66], and Bayesian analyses (BA) were carried out with MrBayes version 3.1.2 [67].

All characters were equally weighted in the MP analyses, and the heuristic search was implemented with 10,000 replicates of random sequence addition, holding 100 trees at each step during stepwise addition, and the tree-bisection-reconnection (TBR) branch-swapping algorithm.

The general time reversible model with a proportion of invariable sites and a discrete gamma distribution for the variable sites (GTR+I+Γ) was chosen based on hierarchical likelihood ratio tests and the Akaike information criterion conducted with Modeltest version 3.7 [68] for ML analyses and with MrModeltest version 2.3 [69] for Bayesian analyses. The model parameters were used to execute a ML heuristic search with 1,000 random addition replicates, holding 2 trees at each step, and applying the TBR algorithm.

To account for the different evolutionary processes occurring at each of the three codon positions, Bayesian analyses were performed with one distinct GTR+I+G model per codon position, with unlinking of base frequencies, GTR, and parameters. Markov chain Monte Carlo (MCMC) sampling was performed for 3,000,000 generations with four simultaneous chains.

The robustness of nodes was assessed by nonparametric bootstrap percentages (BP) after 10,000 pseudoreplicates with 10 random additions for MP using PAUP*4.0b10 [66] and 1,000 pseudoreplicates for ML using PHYML version 2.4.4 [70]. For MP we also calculated the decay index (DI), or Bremer support [71] using the program TreeRot version 3 [72]. Bayesian posterior probabilities (BPP) were calculated from trees that were sampled every 100 generations, after removing the first 5,000 generations as a "burn-in" stage.

As an approximation of minimum values required for a 95% binomial confidence interval for Bootstrap, Decay Index, and Bayesian posterior probabilities, we used calculations obtained from simulations on artificial 4-taxa data sets for internode lengths of 3 through 60 steps [50]. Minimal values for reliability varied from 88 to 100% for BP, from 3 to 15 for DI, and from 91 to 100% for BPP, depending on the branch lengths.

In order to test for two competing hypotheses: monophyly of Erethizontidae including *Chaetomys*, versus monophyly of Echimyidae including *Chaetomys*, we performed the permutation tail probability (T-PTP) [23] test with 100,000 replicates, the Templeton test [24], and the Kishino-Hasegawa (KH) [25] and Shimodaira-Hasegawa (SH) [26] tests.

### Molecular evolutionary rates and molecular dating

To investigate whether a global molecular clock applied to our data, we conducted under PAUP*4.0b10 [66] a likelihood ratio test between log-likelihoods of clock-constrained and non-constrained trees. Estimates of divergence times were calculated using methods based on MCMC Bayesian analyses and non-Bayesian methods. Under Bayesian analyses, dates were estimated either using rates conformed to a molecular clock (CLOC); or using rates uncorrelated, with the rate in each branch independently drawn from a lognormal distribution (UCLN), as described by Rambaut and Drummond [73], both models were implemented in the program BEAST version 1.4.8 [74]. As non-Bayesian methods we used a variant of the nonparametric rate smoothing [75] which compares rates on log scale (NPRS-LOG), and the global rate minimum deformation (GRMD); both methods were implemented in the program Treefinder, version of June 2008 [76].

The methods used to estimate divergence times allowed the incorporation of paleontological constraints into the analyses. As a first calibration point we set the caviomorph radiation in the Late Eocene-Early Oligocene, ca. 34 Ma (Mustersan SALMA – South America Land Mammal Age, as dated by Kay et al. [77], based on the recent discovery of members of Erethizontoidea, Cavioidea, and Octodontoidea superfamilies from the Eocene Santa Rosa local fauna in Amazonian Peru [78]. As a second calibration point we set the octodontoid most recent common ancestor (MRCA) in the Late Oligocene, ca. 27 Ma (Deseadan SALMA) [79]. Finally, as a third calibration point we set the echimyid MRCA in the Early Miocene, ca. 20 Ma (Colhuehuapian SALMA) [80].

## Authors' contributions

RVV conceived the study, carried out the molecular data collection and analyses, and drafted the manuscript. TM performed the karyologic analysis of *Chaetomys subspinosus *and made substantial contributions to the manuscript. VF carried out chromosome preparations of *Chaetomys subspinosus *and made substantial contributions to the manuscript. KV and MJJS carried out chromosome preparations and karyologic analyses of *Euryzygomatomys spinosus*, *Myocastor coypus*, and *Sphigurus villosus *and made substantial contributions to the manuscript. YYY coordinated the study and helped to draft the manuscript. The final manuscript has been read and approved by all authors and all authors take responsibility for the content of the manuscript.

## Supplementary Material

Additional file 1 **List of primers used in the molecular analyses.** Provides a list of the primers used our molecular analyses and each respective sequence, strand and location. Click here for file

## References

[B1] Luckett WP, Hartenberger J-L (1985). Evolutionary relationships among rodents: a multidisciplinary analysis.

[B2] Bryant JD, McKenna MC, Mongolyn Shinzhl*ekh Ukhaany Akademi (1995). Cranial anatomy and phylogenetic position of Tsaganomys altaicus (Mammalia, Rodentia) from the Hsanda Gol Formation (Oligocene), Mongolia.

[B3] Candela AM (2004). A new giant porcupine (Rodentia, Erethizontidae) from the late Miocene of Argentina. Journal of Vertebrate Paleontology.

[B4] Patterson B, Wood AE (1982). Rodents from the Deseadan Oligocene of Bolivia and the relationships of the Caviomorpha.

[B5] Emmons L, Feer F (1997). Neotropical rainforest mammals: a field guide.

[B6] Voss RS, Angermann R, Zoologisches Museum Berlin Germany) (1997). Revisionary notes on neotropical porcupines (Rodentia, Erethizontidae) 1, Type material described by Olfers (1818) and Kuhl (1820) in the Berlin Zoological Museum.

[B7] Bonvicino CR, Penna-Firme V, Braggio E (2002). Molecular and karyologic evidence of the taxonomic status of *Coendou *and *Sphiggurus *(Rodentia: Hystricognathi). J Mammal.

[B8] Nowak RM (1999). Walker's mammals of the world.

[B9] Woods CA, Kilpatrick CW, Wilson DE, Reeder DM (2005). Infraorder Hystricognathi Brandt, 1855. Mammal species of the world: a taxonomic and geographic reference.

[B10] Handley CO, Pine RH (1992). A new species of prehensile-tailed porcupine, genus *Coendou *Lacépède, from Brazil. Mammalia.

[B11] Eisenberg JF, Redford KH (1999). Mammals of the Neotropics.

[B12] Stehlin HG, Schaub S (1951). Die Trigonodontie der simplicidentaten Nager. Schweiz Paläont Abhandl.

[B13] Schaub S (1958). Simplicidentata (= Rodentia). Traité de Paleontologie Edited by Piveteau J Paris: Masson et Cie.

[B14] Woods CA, Wilson DE, Reeder DM (1993). Suborder Hystricognathi. Mammal species of the world: a taxonomic and geographic reference.

[B15] Martin T (1994). On the systematic position of the *Chaetomys subspinosus *(Rodentia: Caviomorpha) based on evidence from the incisor enamel microstructure. J Mamm Evol.

[B16] Carvalho G (2000). Substitution of the deciduous premolar *Chaetomys subspinosus *(Olfers, 1818) (Hystricognathi, Rodentia) and its taxonomic implications Z. Säugetierkunde 65. Z Sauget.

[B17] McKenna MC, Bell SK, Simpson GG (1997). Classification of mammals above the species level.

[B18] Irwin DM, Kocher TD, Wilson AC (1991). Evolution of the cytochrome *b *gene of mammals. J Mol Evol.

[B19] Ma DP, Zharkikh A, Graur D, VandeBerg JL, Li WH (1993). Structure and evolution of opossum, guinea pig, and porcupine cytochrome b genes. J Mol Evol.

[B20] Lara MC, Patton JL, da Silva MN (1996). The simultaneous diversification of South American echimyid rodents (Hystricognathi) based on complete cytochrome *b *sequences. Mol Phylogenet Evol.

[B21] Xia X, Xie Z, Salemi M, Chen L, Wang Y (2003). An index of substitution saturation and its application. Mol Phylogenet Evol.

[B22] Montgelard C, Bentz S, Tirard C, Verneau O, Catzeflis FM (2002). Molecular systematics of Sciurognathi (Rodentia): the mitochondrial cytochrome *b *and 12S rRNA genes support the Anomaluroidea (Pedetidae and Anomaluridae). Mol Phylogenet Evol.

[B23] Faith DP (1991). Cladistic Permutation Tests for Monophyly and Nonmonophyly. Syst Zool.

[B24] Templeton AR, Weir BJ (1983). Convergent evolution and non-parametric inferences from restriction fragment and DNA sequence data. Statistical Analysis of DNA Sequence Data.

[B25] Kishino H, Hasegawa M (1989). Evaluation of the maximum likelihood estimate of the evolutionary tree topologies from DNA sequence data, and the branching order in hominoidea. J Mol Evol.

[B26] Shimodaira H, Hasegawa M (1999). Multiple comparisons of log-likelihoods with applications to phylogenetic inference. Mol Biol Evol.

[B27] Machado T, Silva MJJ, Leal-Mesquita ER, Carmignotto AP, Yonenaga-Yassuda Y (2005). Nine karyomorphs for spiny rats of the genus *Proechimys *(Echimyidae, Rodentia) from North and Central Brazil. Genet Mol Biol.

[B28] Dunnum JL, Salazar-Bravo J, Yates TL (2001). The Bolivian bamboo rat, *Dactylomys boliviensis *(Rodentia: Echimyidae), a new record for chromosome number in a mammal. Z Sauget.

[B29] Aguilera M, Corti M (1994). Craniometric differentiation and chromosomal speciation of the genus *Proechimys *(Rodentia, Echimyidae). Z Sauget.

[B30] Leite YLR, University of California Berkeley. Museum of Vertebrate Zoology (2003). Evolution and systematics of the Atlantic tree rats, genus Phyllomys (Rodentia, Echimyidae) with description of two new species.

[B31] Benirschke K (1968). The chromosome complement and meiosis of the North American porcupine. J Hered.

[B32] Concepción JL, Molinari J (1991). *Sphiggurus vestitus pruinosus *(Mammalia, Rodentia, Erethizontidae): the karyotype and its phylogenetic implications, descriptive notes. Stud Neotrop Fauna Environm.

[B33] Bonvicino CR, Almeida FC, Cerqueira R (2000). The karyotype of *Sphiggurus villosus *(Rodentia: Erethizontidae) from Brazil. Stud Neotrop Fauna Environm.

[B34] George W, Weir BJ (1974). Hystricomorph chromosomes. Symp Zool Soc Lond.

[B35] Lima FS (1994). Cariótipos em espécies de Dasyproctidae e Erethizontidae, com discussão da evolução cromossômica (Rodentia, Caviomorpha). Braz J Genet Supplement.

[B36] Reig OA, Useche M (1976). [Karyotype diversity and systematics in Venezuelan populations of *Proechimys *(Rodentia, Echimyidae) with additional information of Peruvian and Colombian populations]. Acta Cient Venez.

[B37] Leal-Mesquita ER (1991). Estudos citogenéticos em dez espécies de roedores brasileiros da família Echimyidae. Dissertation (Master).

[B38] Hsu TC, Benirschke K (1977). An Atlas of mammalian chromosomes.

[B39] George W, Luckett WP, Hartenberger J-L (1985). Reproductive and chromosomal characters of ctenodactylids as a key to their evolutionary relationships. Evolutionary relationships among rodents: a multidisciplinary analysis.

[B40] O'Brien SJ, Menninger JC, Nash WG (2006). Atlas of mammalian chromosomes.

[B41] Gallardo MH, Gonzalez CA, Cebrian I (2006). Molecular cytogenetics and allotetraploidy in the red vizcacha rat, *Tympanoctomys barrerae *(Rodentia, Octodontidae). Genomics.

[B42] Brandt JF (1855). Beitrage zur nahern Kenntniss der Saugetiere Russlands. Mem Acad Imp St Petersbourg.

[B43] Nedbal MA, Allard MW, Honeycutt RL (1994). Molecular systematics of hystricognath rodents: evidence from the mitochondrial 12S rRNA gene. Mol Phylogenet Evol.

[B44] Marivaux L, Vianey-Liaud M, Jaeger J-J (2004). High-level phylogeny of early Tertiary rodents: dental evidence. Zool J Linn Soc.

[B45] Catzeflis FM, Hanni C, Sourrouille P, Douzery E (1995). Molecular systematics of hystricognath rodents: the contribution of sciurognath mitochondrial 12S rRNA sequences. Mol Phylogenet Evol.

[B46] Huchon D, Douzery EJ (2001). From the Old World to the New World: a molecular chronicle of the phylogeny and biogeography of hystricognath rodents. Mol Phylogenet Evol.

[B47] Poux C, Chevret P, Huchon D, de Jong WW, Douzery EJ (2006). Arrival and diversification of caviomorph rodents and platyrrhine primates in South america. Syst Biol.

[B48] Sarich VM, Cronin JE, Ciochon RI, Chiarelli AB (1980). South American mammal molecular systematics, evolutionary clocks, and continental drift. Evolutionary Biology of the New World Monkeys and Continental Drift.

[B49] Hugot JP, Page RDM (2002). New evidence of hystricognath rodents monophyly from the phylogeny of their pinworms. Tangled trees: phylogeny, cospeciation, and coevolution.

[B50] Zander RH (2004). Minimal Values for Reliability of Bootstrap and Jackknife Proportions, Decay Index, and Bayesian Posterior Probability. PhyloInformatics.

[B51] Huchon D, Catzeflis FM, Douzery EJ (2000). Variance of molecular datings, evolution of rodents and the phylogenetic affinities between Ctenodactylidae and Hystricognathi. Proc Biol Sci.

[B52] Huchon D, Catzeflis FM, Douzery EJ (1999). Molecular evolution of the nuclear von Willebrand factor gene in mammals and the phylogeny of rodents. Mol Biol Evol.

[B53] Leite YL, Patton JL (2002). Evolution of South American spiny rats (Rodentia, Echimyidae): the star-phylogeny hypothesis revisited. Mol Phylogenet Evol.

[B54] Adkins RM, Walton AH, Honeycutt RL (2003). Higher-level systematics of rodents and divergence time estimates based on two congruent nuclear genes. Mol Phylogenet Evol.

[B55] Marivaux L, Vianey-Liaud M, Welcomme J-L, Jaeger J-J (2002). The role of Asia in the origin and diversification of hystricognathous rodents. Zool Scr.

[B56] Honeycutt RL, Rowe DL, Gallardo MH (2003). Molecular systematics of the South American caviomorph rodents: relationships among species and genera in the family Octodontidae. Mol Phylogenet Evol.

[B57] Gallardo MH, Kirsch JAW (2001). Molecular Relationships Among Octodontidae (Mammalia: Rodentia: Caviomorpha). J Mamm Evol.

[B58] Opazo JC (2005). A molecular timescale for caviomorph rodents (Mammalia, Hystricognathi). Mol Phylogenet Evol.

[B59] Galewski T, Mauffrey JF, Leite YL, Patton JL, Douzery EJ (2005). Ecomorphological diversification among South American spiny rats (Rodentia; Echimyidae): a phylogenetic and chronological approach. Mol Phylogenet Evol.

[B60] Fetzner JW (1999). Extracting high-quality DNA from shed reptile skins: a simplified method. Biotechniques.

[B61] Hall ER (1981). The mammals of North America.

[B62] Jeanmougin F, Thompson JD, Gouy M, Higgins DG, Gibson TJ (1998). Multiple sequence alignment with Clustal X. Trends Biochem Sci.

[B63] Maddison DR, Maddison WP (2005). MacClade, version 4.08.

[B64] Tamura K, Dudley J, Nei M, Kumar S (2007). MEGA4: Molecular Evolutionary Genetics Analysis (MEGA) software version 4.0. Mol Biol Evol.

[B65] Xia X, Xie Z (2001). DAMBE: software package for data analysis in molecular biology and evolution. J Hered.

[B66] Swofford DL (2003). PAUP*. Phylogenetic Analysis Using Parsimony (* and Other Methods), version 4b10.

[B67] Ronquist F, Huelsenbeck JP (2003). MrBayes 3: Bayesian phylogenetic inference under mixed models. Bioinformatics.

[B68] Posada D, Crandall KA (1998). MODELTEST: testing the model of DNA substitution. Bioinformatics.

[B69] Nylander JAA (2004). MrModeltest. MrModeltest, version 2 Program distributed by the author.

[B70] Guindon S, Gascuel O (2003). A simple, fast, and accurate algorithm to estimate large phylogenies by maximum likelihood. Syst Biol.

[B71] Bremer K (1994). Branch support and tree stability. Cladistics.

[B72] Sorenson MD, Franzosa EA (2007). TreeRot, version 3.

[B73] Drummond AJ, Ho SY, Phillips MJ, Rambaut A (2006). Relaxed phylogenetics and dating with confidence. PLoS Biol.

[B74] Drummond AJ, Rambaut A (2007). BEAST: Bayesian evolutionary analysis by sampling trees. BMC Evol Biol.

[B75] Sanderson MJ (1997). A Nonparametric Approach to Estimating Divergence Times in the Absence of Rate Constancy. Mol Biol Evol.

[B76] Jobb G, von Haeseler A, Strimmer K (2004). TREEFINDER: a powerful graphical analysis environment for molecular phylogenetics. BMC Evol Biol.

[B77] Kay RF, Madden RH, Vucetich MG, Carlini AA, Mazzoni MM, Re GH, Heizler M, Sandeman H (1999). Revised geochronology of the casamayoran south american land mammal age: climatic and biotic implications. Proc Natl Acad Sci USA.

[B78] Frailey CD, Campbell KE, Campbell Jr KE (2004). The Rodents of the Santa Rosa Local Fauna. The Paleogene Mammalian Fauna of Santa Rosa, Amazonian Peru.

[B79] Vucetich MG, Verzi DH, Hartenberger J-L (1999). Review and analysis of the radiation of the South American Hystricognathi (Mammalia, Rodentia). C R Acad Sci Paris.

[B80] Carvalho GAS, Salles LO (2004). Relationships among extant and fossil echimyids (Rodentia: Hystricognathi). Zool J Linn Soc.

[B81] Allard MW, Honeycutt RL (1992). Nucleotide sequence variation in the mitochondrial 12S rRNA gene and the phylogeny of African mole-rats (Rodentia: Bathyergidae). Mol Biol Evol.

[B82] Walton AH, Nedbal MA, Honeycutt RL (2000). Evidence from intron 1 of the nuclear transthyretin (Prealbumin) gene for the phylogeny of African mole-rats (Bathyergidae). Mol Phylogenet Evol.

[B83] Rowe DL, Honeycutt RL (2002). Phylogenetic relationships, ecological correlates, and molecular evolution within the cavioidea (mammalia, rodentia). Mol Biol Evol.

[B84] Spotorno AE, Valladares JP, Marin JC, Zeballos H (2004). Diversidad molecular entre cuyes domésticos (*Cavia porcellus*) y su relación filogenética cercana con la especie silvestre andina *Cavia tschudii*. Rev Chil Hist Nat.

[B85] Faulkes CG, Bennett NC, Bruford MW, O'Brien HP, Aguilar GH, Jarvis JU (1997). Ecological constraints drive social evolution in the African mole-rats. Proc Biol Sci.

[B86] Faulkes CG, Abbott DH, O'Brien HP, Lau L, Roy MR, Wayne RK, Bruford MW (1997). Micro- and macrogeographical genetic structure of colonies of naked mole-rats *Heterocephalus glaber*. Mol Ecol.

[B87] da Silva MN, Patton JL (1993). Amazonian phylogeography: mtDNA sequence variation in arboreal echimyid rodents (Caviomorpha). Mol Phylogenet Evol.

[B88] Slamovits CH, Cook JA, Lessa EP, Rossi MS (2001). Recurrent amplifications and deletions of satellite DNA accompanied chromosomal diversification in South American tuco-tucos (genus *Ctenomys*, Rodentia: Octodontidae): a phylogenetic approach. Mol Biol Evol.

[B89] Lessa EP, Cook JA (1998). The molecular phylogenetics of tuco-tucos (genus *Ctenomys*, Rodentia: Octodontidae) suggests an early burst of speciation. Mol Phylogenet Evol.

