# Supplementary material for: The taxonomic status of the endangered thin-spined porcupine, Chaetomys subspinosus (Olfers, 1818), based on molecular and karyologic data
Source: BMC Evol Biol. 2009 Feb 3;9:29. doi: 10.1186/1471-2148-9-29 (PMC2646700; doi:10.1186/1471-2148-9-29)
Supplement: Additional file 1 — List of primers used in the molecular analyses. Provides a list of the primers used our molecular analyses and each respective sequence, strand and location. [file 1471-2148-9-29-S1.pdf]

List of primers used in the molecular analyses.

Primer name	Primer sequence (5’ to 3’)	Strand	3’-end
MVZ 51	CTA GAT CCC CAA GTY TCA GRA TA	L	13.833
MVZ 05	CGA AGC TTG ATA TGA AAA ACC ATC GTT G	L	14.115
MVZ 127	TRY TAC CAT GAG GAC AAA TAT C	L	14.554
MVZ 16	AAA TAG GAA RTA TCA YTC TGG TTT RAT	H	14.940
MVZ 108	CCA ATG TAA TTT TTA TAC	H	15.292
MVZ 14	GGT CTT CAT CTY HGG YTT ACA AGA C	H	15.309

The 3’-end refers to the position in Mus. L, light strand; H, heavy strand. MVZ, Museum of Vertebrate Zoology, University of California, Berkeley.
